# Usefulness of the ratio total IgE / specific IgE (sIgE) to predict tolerance in children allergic to cow's milk proteins (CMP)

**DOI:** 10.1186/2045-7022-3-S3-P101

**Published:** 2013-07-25

**Authors:** A Machinena, M Alvaro, J Lozano, M Piquer, O Dominguez, AM Plaza, MT Giner

**Affiliations:** 1 Pediatric Allergy and Clinical Immunology Section, Hospital Sant Joan de Déu. Universitat de Barcelona, Esplugues (Barcelona), Spain

## Background

Cow's milk (CM) IgE-mediated allergy is the most common food allergy in children. It affects 2 - 3% of the general population. About 80% of children allergic to CMP reach natural tolerance in the third year of life. It remains difficult to decide when it is time to attempt a controlled food challenge (CFC) to reintroduce CM in their diets. Objectives. To evaluate if the ratio total IgE / CM sIgE or total IgE / casein sIgE can be more useful than the single measurement of sIgE to predict tolerance in our patients, and to determine the highest sensitivity cut-off point.

## Methods

Retrospective, observational study. The medical records of 178 patients allergic to CM with CFC performed between January 2010 and December 2011 were reviewed. Clinical data, skin prick tests (SPT), total IgE, CM sIgE and casein sIgE at baseline and at the time of the CFC were recorded. These measures were compared between groups of tolerant and non-tolerant patients using the nonparametric Mann-Whitney. ROC curves were performed to evaluate predictive values of tolerance for total IgE, CM sIgE, casein sIgE and ratios (total IgE / CM sIgE; total IgE / casein sIgE).

## Results

178 patients were included. 53% boys, 47% girls; mean age 4 years (range: 5 months - 13 years) at CFC. 107 patients (60%) tolerated, while 71 (40%) continued to be allergic. Of these 71 children, 6 (8%) had moderate anaphylaxis, 30 (42%) mild anaphylaxis, 15 (21%) urticaria and 7 patients (10%) rhinitis. Mean value for CM sIgE 19.12 KU / L (range: 0-727) and for casein sIgE 19.7 KU / L (range: 0-867). CM sIgE < 2.5 KU/L is a good marker for predicting tolerance but the ratio total IgE / CM sIgE ratio ≥ 3.75 allows, with greater reliability, the detection of tolerant patients. If only CM sIgE values < 2.5 kU/L had been used, 22% of our patients wouldn’t have undergone a CFC which has been successful.

## Conclusion

The use of the ratio total IgE / CM sIgE is useful for predicting tolerance in CM allergic patients, avoiding, thus, unnecessary exclusion diets.

## Disclosure of interest

None declared.

